# MMP9 overexpression is associated with good surgical outcome in children with UPJO: Preliminary results

**DOI:** 10.1186/s12894-016-0162-6

**Published:** 2016-07-23

**Authors:** Sabrina Thalita Reis, Kátia R. M. Leite, Nayara Izabel Viana, Roberto Iglesias Lopes, Caio Martins Moura, Renato F. Ivanovic, Marcos Machado, Francisco Tibor Denes, Amilcar Giron, William Carlos Nahas, Miguel Srougi, Carlo C. Passerotti

**Affiliations:** Urology Department, Laboratory of Medical Investigation (LIM55), University of Sao Paulo Medical School, Av. Dr. Arnaldo 455, 2° floor, room 2145, 01246-903 Sao Paulo, Brazil

**Keywords:** UPJO, Metaloproteinases, Children

## Abstract

**Background:**

Ureteropelvic junction obstruction (UPJO) diagnosed prenatally occurs in 1:150 – 1:1200 pregnancies. Although many studies investigating the molecular changes of this obstructed segment have been performed, the underlying mechanisms are still unclear. The role of extracellular matrix (ECM) components remains controversial, and the investigations in the field of ECM changes, might help the better understanding of the pathogenesis of this common condition. The aim of the present study was to investigate for the first time in the literature whether MMP9 and its specific inhibitors, TIMP1 and RECK, are expressed in a reproducible, specific pattern in UPJ.

**Methods:**

UPJO specimens were obtained from 16 children at the time of dismembered pyeloplasty due to intrinsic UPJ stenosis. Expression levels of the three genes (MMP9, TIMP1 and RECK) were analyzed by quantitative real-time polymerase chain reaction (qRT-PCR). Then correlated the expression levels of the genes according to grade study population that was divided in 2 categories according to Society of Fetal Urology classification, grade 3 (moderate) and 4 (severe). For DTPA we subdivided the childrens in 2 groups, obstructive (T 1/2 more than 20 min) and partial obstructive (T 1/2 between 10 and 20 min) and success in a surgery was defined as decrease in T 1/2 to less than 20 min, absence of symptoms, improving renal function and decreasing dilatation on successive exams.

**Results:**

MMP9 was underexpressed and TIMP1 and RECK were overexpressed in children with obstructive DTPA but the differences were not statistically significant. Overexpression of MMP9 was higher among patients with severe grade of UPJ compared to those with moderate grade. Surprisingly expression levels of MMP-9 was three times higher in children who were successfully treated by surgery (*n* = 10) (*p* = 0.072), so those who were followed for at least 1 year after surgery and remained with improvement in renal function and decreasing dilation on intravenous urogram and TIMP-1 was underexpressed in 100 % of this cases (*p* = 0.00).

**Conclusions:**

We showed an increase in expression of MMP9 and a decrease in expression of TIMP1 in children who improving renal function and decreasing dilation after surgery. We believe that the higher expression of MMP9 in these cases can reflect an increase in degradation and remodeling process that could be used as a marker for surgical outcome.

## Background

Ureteropelvic junction obstruction (UPJO) diagnosed prenatally occurs in 1:150–1:1200 pregnancies [1, 2]. Hydronephrosis can be a result of anatomical or functional obstruction. It is diagnosed almost twice as often in boys and can affect either one (most often the left) or both kidneys [3–7].

Although many studies investigating the molecular changes of this obstructed segment have been performed, the underlying mechanisms are still unclear. The studies revealed abnormal innervation patterns and abnormalities of smooth muscle or collagen composition as well as reduction in the number of interstitial cells of Cajal [8, 9]. The role of extracellular matrix (ECM) components and neural distribution remains controversial, and the investigations in the field of ECM changes, and innervation patterns might help the better understanding of the pathogenesis of this common condition.

ECM is a biologically active composition of structural and adhesive proteins embedded in a hydrated ground substance of glycosaminoglycans and proteoglycans. Matrix metalloproteinases (MMPs) are secreted from connective tissue cells of mesenchymal origin, part of a family of closely related proteolytic enzymes, able to degrade most component of the ECM [10]. The MMPs are proteolytic enzymes present in both normal and pathologic tissues in which matrix remodeling occurs. The gelatinase (MMP9) degrades the basal membrane. Studies have explored the role of ECM in primary obstructed and primary refluxing megaureters showing both a decrease in smooth muscle and an increase in the ECM [11], and higher ECM turnover in refluxing ureteral endings [12]. Early descriptions of the UPJ alterations were those of extensive fibrosis and muscular attenuation. Innervation abnormalities at the UPJ have been suggested as another cause for UPJ obstruction, based on findings of reduced nerve distribution at this location [13].

The aim of the present study was to investigate whether MMP9 and its specific inhibitors, Tissue inhibitors of metalloproteinases 1 (TIMP1) and Reversion-inducing cysteine-rich protein with Kazal motifs (RECK), are expressed in a reproducible, specific pattern in UPJO. Additionally, we evaluated the correlation between the expression of these genes and important clinical parameters (Grade and DTPA) and surgical outcome in childrens with UPJ submitted to pyeloplasty.

## Methods

### Patient selection

After obtaining institutional ethic board approval, UPJ specimens were obtained from 16 children (11 boys, 5 girls) at the time of dismembered pyeloplasty due to intrinsic UPJ stenosis (median age, 4.0 years, range, 1–16 years). All cases of UPJ obstruction were confirmed on the basis of radiological, scintigraphic, and operative findings.

We first analyzed MMP9, TIMP1 and RECK expression levels in fresh tissue specimens from the 16 children using quantitative real-time polymerase chain reaction (qRT-PCR). Tissue sample was taken at the time of the surgery and a small fragment of 1 by 1 cm from the transition between ureter and pelvis were analysed.

Then correlated the expression levels of the genes with important clinical parameters (Grade and DTPA) and surgical outcome in children with UPJO. According to grade study population was divided in 2 categories according to Society of Fetal Urology classification, grade 3 (moderate) and 4 (severe). For DTPA we subdivided the childrens in 2 groups, obstructive (T 1/2 more than 20 min) and partial obstructive (T 1/2 between 10 and 20 min) and success in a surgery is defined as decrease in T 1/2 to less than 20 min, absence of symptoms, improving renal function and decreasing dilatation on successive exams.

### RNA isolation and cDNA synthesis

All tissue samples were obtained from surgical specimens and immediately frozen at −170 °C in liquid nitrogen. Total RNA was isolated with an RNA aqueous Kit (Applied Biosystems, CA, USA) according to the manufacturer’s instructions. RNA concentration was determined by 260/280 nM absorbance using a Nanodrop ND-1000 spectrophotometer (Thermo Scientific). cDNA was generated using a High Capacity cDNA Reverse Transcription Kit (Applied Biosystems, CA, USA). The reactions were incubated at 25 °C for 10 min, followed by 37 °C for 120 min and 85 °C for 5 min. The cDNA was stored at −20 °C until further use.

### Quantitative real-time PCR and gene expression

Expression levels of the three genes were analyzed by qRT-PCR using an ABI 7500 Fast Real-Time PCR System (Applied Biosystems). Target sequences were amplified in a 10-μl reaction containing 5 μl of TaqMan Universal PCR Master Mix, 0.5 μl of TaqMan Gene Expression Assays (primers and probes; see Table 1), 1 μl of cDNA and 3.5 μl of DNase-free water. The PCR cycling conditions were 2 min at 50 °C, 10 min at 95 °C, and then 40 cycles of 15 s at 95 °C and 1 min at 60 °C. A TaqMan B2M assay was utilized as the endogenous control (Table 1).

**Table 1:** Primers utilized

Gene symbol	Assays ID
MMP9	Hs00957562_m1
TIMP1	Hs00212624_m1
RECK	Hs01019179_m1
B2M	Hs99999907_m1

### Statistical analysis

We used the ΔΔCT method to calculate the relative expression of the three target genes using the formula ΔΔCT. The fold change in gene expression was calculated as 2^-ΔΔCT^. To compare the clinical characteristics of children with UPJ, we used the Mann–Whitney test. Statistical analysis was performed using SPSS 19.0 for Windows using a significance of *p* ≤0.05.

## Results

**Fig. 1:**
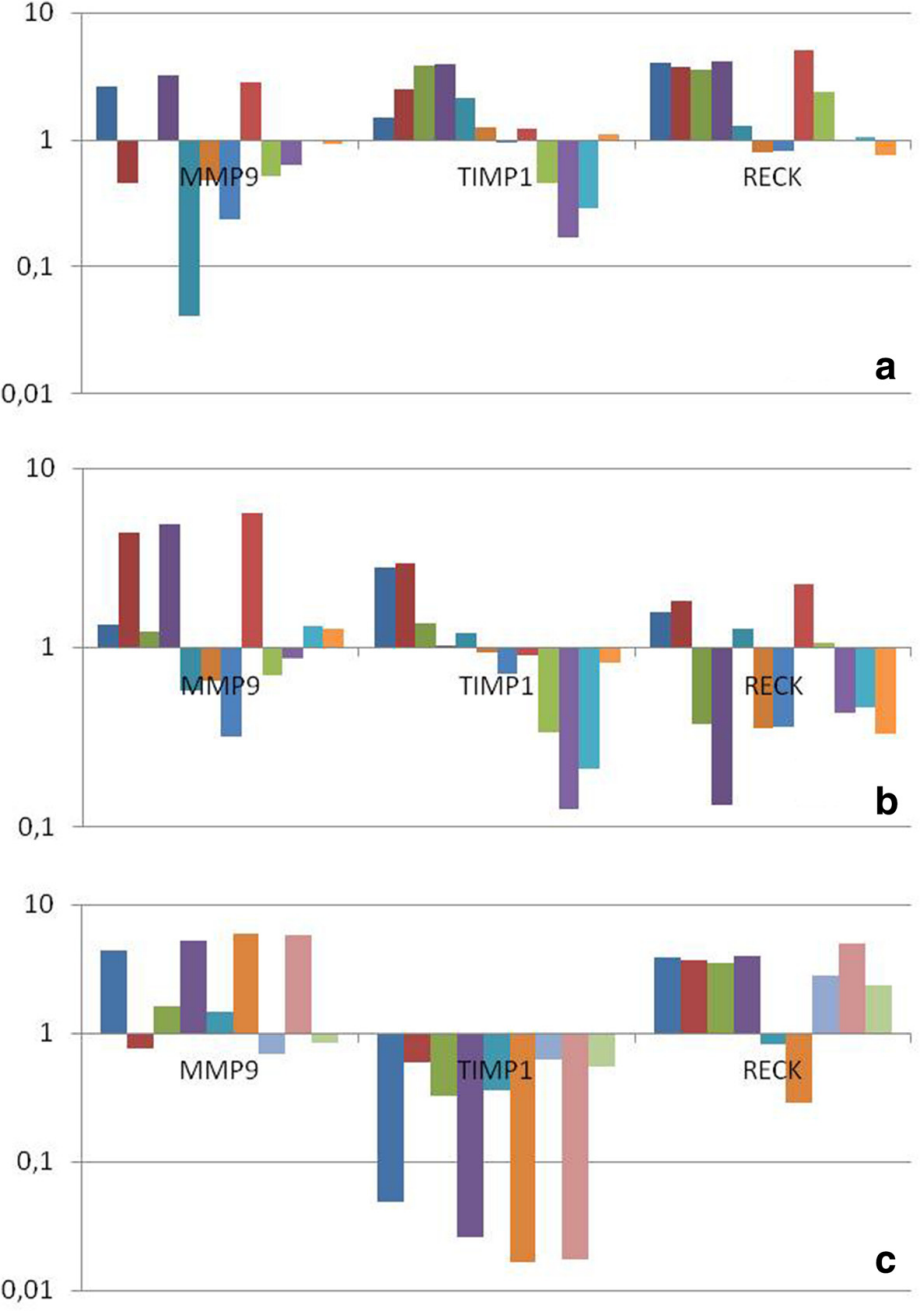
Expression profile of MMP9, TIMP1 and RECK according to DTPA (**a**), grade (**b**) and success of treatment (**c**) in tissue of UPJ children

The children were grouped according to DTPA, grade and success of treatment. 13 children with obstructive DTPA were compared to 3 children with a partial obstruction, and as demonstrated in Fig. 1a we showed that MMP9 was underexpressed and TIMP1 and RECK were overexpressed in children with obstructive DTPA but the differences were not statistically significant (MMP9, *p* = 0.170; TIMP1, *p* = 0.389; RECK, *p* = 0.389).

Our analysis of MMP9, TIMP1 and RECK expression levels according to grade of obstruction is shown in Fig. 1b. Overexpression of MMP9 was higher among patients with severe grade of UPJ (*n* = 12) compared to those with moderate grade (*n* = 4). However, statistical analysis revealed that these differences were not significant (*p* = 0.694). TIMP1 and RECK did not significantly differ considering grade of UPJ (TIMP-1, *p* = 0.684, RECK, *p* = 0.684).

When MMP9, TIMP1 and RECK expression levels were analyzed according to surgical outcome, surprisingly we found that the median expression levels of MMP-9 was three times higher in children who were successfully treated by surgery (*n* = 10) compared to those did not have success (*n* = 6), with a marginal significance (*p* = 0.072), TIMP-1 was underexpressed in 100 % of this cases (*p* = 0.00) and RECK was overexpressed in 80 % of this same cases (*p* = 0.082). This results is shown in Fig. 1c.

### Discussion

UPJ obstruction is mostly considered as a functional obstruction originating from abnormalities in the smooth muscle of the pelvis and ureter [14]. Although surgery in UPJ obstruction is efficient to protect the patient against renal function lost, results obtained in both experimental and human-studies suggest that UPJ obstruction induces permanent modifications of the renal parenchyma.

The nature of the abnormalities at the UPJ in children with congenital intrinsic UPJ obstruction remains controversial. Many studies revealed that UPJ obstruction is associated with a significant difference in the collagen and smooth muscle structural components [15]. The finding of the increased tissue matrix ratio was believed to decrease the ureteral distensibility resulting in damage to muscle cells influencing the contractility [16]. Furthermore, a variety of intrarenal factors lead to progressive interstitial and renal parenchyma fibrosis in patients with Congenital anomalies of the kidney and urinary tract, like UPJ, including growth factors, cytokines, chemokines and adhesion molecules, which are produced by the hydronephrotic kidney. An altered renal expression of growth factors and cytokines modulates cell death by apoptosis or phenotypic transition of glomerular, tubular, and vascular cells. Mediators of cellular injury include hypoxia, ischemia, and reactive oxygen species, while fibroblasts undergo myofibroblast transformation with increased deposition of extracellular matrix [17].

The present study is the first to investigate the expression of MMP9 and its negative controllers in obstructed UPJ tissue. The change in expression of ECM components could be an alternative mechanism leading to UPJ obstruction together with the reduction of interstitial cells of Cajal. The reduction in peristalsis, result of the reduced number of Cajal’s cells associated to reduction in distensibility, result of alteration in ECM components could be the physiopathology of the disease [18]. MMPs have many important functions in wound healing processes and angiogenesis. In the case of deregulation of their production, matrix degradation and turnover are the consequences [19] and It has been shown that an increase in ECM turnover influences the neuronal network within the ureteral wall. Also, some MMPs have been proved to be neurotoxic degrading ECM proteins like collagen type 1, which are normally able to protect cultured neurons from cytotoxic cell death [20].

Defective collagen production from smooth muscle cells has been held responsible for this pathology and decreased neural cells have been thought to play an important role, especially in intrinsic type obstructions [21]. There are other studies showing that this disintegration in the configuration of smooth muscle and overdeposition of collagen may be an etiologic factor, and collagen to smooth muscle ratio may have a prognostic value [15].

There are some limitation that should be pointed out. Our small number of cases would interfere in our findings and may affect in our subgroups analysis (successful against failure and intrinsic against crossing vessel group). Since it refers to a low incidence disease with decreasing surgical indication it may be of difficult to increase sample size in a small period of time. Also the lack of control group because of the absence normal tissue also may impact in our findings. At last, there is an intentional bias in selection, including a higher number of failure cases does not reflects the overall success in the surgical approach.

## Conclusion

Here, curiously we showed an increase in expression of MMP9 and a decrease in expression of TIMP1 in children who were successfully treated by surgery. UPJ obstruction is associated with a significant difference in the collagen, and MMPs are potent proteolytic enzymes that are known to play a key role in the degradation of this component, therefore, we believe that the higher expression of MMP9 in these cases can reflect an increase in degradation of collagen and remodeling process that could be used as a marker for surgical outcome.

## Abbreviations

cDNA, complementary deoxyribonucleic *acid*; ECM, extracellular matrix; MMP, matrix metalloproteinase; qRT-PCR, quantitative real-time polymerase chain reaction; RECK, reversion-inducing cysteine-rich protein with Kazal motif; *RNA,* ribonucleic *acid*; TIMP-1, tissue inhibitor of metalloproteinases 1; UPJO, ureteropelvic junction obstruction
